# Supplementary material for: A high-resolution mRNA expression time course of embryonic development in zebrafish
Source: eLife. 2017 Nov 16;6:e30860. doi: 10.7554/eLife.30860 (PMC5690287; doi:10.7554/eLife.30860)
Supplement: Supplementary file 6. [file elife-30860-supp6.zip › biolayout-clusters-files/Cluster155-genes.html]

Cluster155


# Cluster155: Genes

| Ensembl ID | Gene Name | Chr | Start | End | Biotype |
| --- | --- | --- | --- | --- | --- |
| ENSDARG00000098967 | RABGAP1 | 10 | 8704645 | 8815033 | protein\_coding |
| ENSDARG00000062272 | afg3l2 | 2 | 21794687 | 21811838 | protein\_coding |
| ENSDARG00000059244 | arfip2a | 15 | 32391083 | 32409327 | protein\_coding |
| ENSDARG00000079555 | ccdc85cb | 20 | 5113850 | 5184929 | protein\_coding |
| ENSDARG00000002494 | itgb6 | 11 | 10991789 | 11035637 | protein\_coding |
| ENSDARG00000056075 | rca2.1 | 23 | 26167940 | 26193747 | protein\_coding |
| ENSDARG00000035181 | slc25a46 | 5 | 55250675 | 55263203 | protein\_coding |
| ENSDARG00000040463 | slc25a51b | 14 | 4071257 | 4077723 | protein\_coding |
| ENSDARG00000079097 | tanc2a | 3 | 22447822 | 22684511 | protein\_coding |
